# Reusable Ag@TiO_2_-Based Photocatalytic Nanocomposite Membranes for Solar Degradation of Contaminants of Emerging Concern

**DOI:** 10.3390/polym13213718

**Published:** 2021-10-28

**Authors:** Lamine Aoudjit, Hugo Salazar, Djamila Zioui, Aicha Sebti, Pedro Manuel Martins, Senentxu Lanceros-Mendez

**Affiliations:** 1 Unité de Développement des Équipementssolaires, UDES/Centre de Développement des Energies Renouvelables, CDER, Bou Ismail, W. Tipaza 42415, Algéria; lamineaoudjit@yahoo.fr (L.A.); ziouidjamila@yahoo.fr (D.Z.); sebti.aicha@udes.dz (A.S.); 2 Centre/Department of Physics, Campus de Gualtar, University of Minho, 4710-057 Braga, Portugal; hsalazar@fisica.uminho.pt; 3 Centre/Department of Chemistry, Campus de Gualtar, University of Minho, 4710-057 Braga, Portugal; 4 Institute of Science and Innovation on Bio-Sustainability (IB-S), University of Minho, 4710-057 Braga, Portugal; 5 Centre of Molecular and Environmental Biology, Campus de Gualtar, University of Minho, 4710-057 Braga, Portugal; 6 BCMaterials, Basque Centre for Materials, Applications and Nanostructures, UPV/EHU Science Park, 48940 Leioa, Spain; 7 IKERBASQUE, Basque Foundation for Science, 48013 Bilbao, Spain

**Keywords:** ANFIS, metronidazole, micropollutants, nanocomposite membrane, photocatalysis, water remediation

## Abstract

Two significant limitations of using TiO_2_ nanoparticles for water treatment applications are reduced photocatalytic activity under visible radiation and difficulty recovering the particles after use. In this study, round-shaped Ag@TiO_2_ nanocomposites with a ≈21 nm diameter and a bandgap energy of 2.8 eV were synthesised by a deposition-precipitation method. These nanocomposites were immobilised into a porous poly (vinylidene fluoride-hexafluoropropylene) (PVDF-HFP) matrix and well-distributed within the pores. The photocatalytic activity of Ag@TiO_2_/PVDF-HFP against metronidazole (MNZ) under solar radiation was evaluated. Further, an adaptive neuro-fuzzy inference system (ANFIS) was applied to predict the effect of four independent variables, including initial pollutant concentration, pH, light irradiation intensity, and reaction time, on the photocatalytic performance of the composite membrane on MNZ degradation. The 10% Ag@TiO_2_/PVDF-HFP composite membrane showed a maximum removal efficiency of 100% after 5 h under solar radiation. After three use cycles, this efficiency remained practically constant, demonstrating the membranes’ reusability and suitability for water remediation applications.

## 1. Introduction

The extensive use of pharmaceuticals and personal care products leads to serious human health and environmental problems [1]. These compounds have been described as contaminants of emerging concern (CEC) due to their harmful effects on aquatic organisms and humans [2]. Inadequate control over these pollutants contributes to their spread in aquatic ecosystems worldwide [3].

Among contaminants, antibiotics are the most concerning ones, as they are intensively used to fight human and animal diseases in cattle raising and agriculture [4]. After its use, the non-metabolised part of an antibiotic is excreted into wastewater by urine and faeces. A dramatic 67% increase in antibiotics intake is expected worldwide until 2030 [5].

Metronidazole (MNZ) is an antibiotic that belongs to the 5-nitroimidazole family, defined by the World Health Organization as an “essential medicine”, with widespread use in the treatment of infections caused by anaerobic bacteria and protozoa [6,7,8]. Due to its low biodegradability and high solubility in water, MNZ can easily be present in high concentrations in aquatic ecosystems [9,10]. The high MNZ concentration, alongside its carcinogenic, mutagenic, and cytotoxic properties, may cause severe diseases such as encephalopathies [11,12]. It has been shown that even in treated wastewater MNZ removal is not complete, showing the inefficiency of conventional wastewater treatment methods [13,14]. In this context, the research for materials and technologies to efficiently remove CECs before the discharge into water bodies is necessary.

Among different technologies, photocatalysis is considered a promising approach to remove MNZ and other persistent contaminants from water [15]. In the photocatalytic process, pollutants are oxidised by reactions with free radicals generated by photocatalysts. Titanium dioxide (TiO_2_) is the utmost used photocatalyst, as it presents a high photocatalytic activity, large surface area, reduced toxicity, low cost, and excellent thermal as well as chemical stability [16,17]. Despite the efficient degradation rates, pristine TiO_2_ nanoparticles present limitations, such as reduced photocatalytic activity under visible radiation and a high recombination of electron/hole pairs [18]. Different approaches have been employed to circumvent these limitations, such as doping [19], functionalisation with noble metals [20], and the development of hybrid materials [21]. In this scope, the functionalisation of TiO_2_ with metals such as Co [22], Cu [23], Au [24], and Ag [25] has been applied to introduce intermediate energy levels and reduce the bandgap of TiO_2_ since these metals, when irradiated, are able to receive electrons and prevent the recombination of electron/hole pairs [26]. The development of silver (Ag)/TiO_2_ nanoparticles improves visible radiation absorption, favouring photocatalytic efficiency [27].

Another limitation of TiO_2_ nanoparticles in suspension is their cumbersome recyclability, which implies filtration/separation processes that are time-consuming and expensive. Additionally, the use of slurry systems unavoidably causes the release of photocatalytic nanoparticles into water bodies, causing secondary pollution and deleterious effects on organisms [28]. Thus, in recent decades, significant research has been carried out regarding TiO_2_ immobilisation into polymeric substrates, whose pore size and porosity can be controlled [29].

Among several substrates, poly (vinylidene fluoride) (PVDF) and co-polymers arise as engaging materials for membrane applications based on their exceptional chemical, UV, mechanical, and thermal stability [17,30,31]. Poly (vinylidene fluoride-hexafluoropropylene), PVDF-HFP, is a co-polymer of PVDF, widely used in different applications, including energy harvesting [32] and water remediation [33]. This polymer is easily processable in various morphologies, including porous membranes, thin films, and fibre mats [34]. The HFP groups increase the free volume, solubility, mechanical strength, and hydrophobicity of and decrease PVDF’s crystallinity of PVDF-HFP [35].

As the photocatalytic process is somewhat affected by different parameters, simulating and modelling based on conventional mathematical approaches are quite complicated [36]. In this context, an adaptive neuro-fuzzy inference system (ANFIS) allows for the prediction of water quality parameters, as has been previously demonstrated [37,38]. Additionally, the application of an ANFIS model has been reported to evaluate wastewater treatment processes, namely in the elimination of organic dyes [39,40], oily wastewater [41], chemical additives [36], antibiotics [42] and heavy metals [43,44].

In this work, 10% Ag@TiO_2_/PVDF-HFP composite membranes were produced and characterised for the photocatalytic degradation of MNZ under solar radiation. The analysis was carried out to understand how the 10% Ag@TiO_2_/PVDF-HFP nanocomposite performance is affected by operating parameters, such as pH, MNZ initial concentration, and light radiation intensity. Simultaneously, the ANFIS model was used to evaluate the prepared composite membrane’s performance in the pollutant degradation process, depending on the operating parameters.

## 2. Materials and Methods

### 2.1. Materials

Metronidazole (MNZ-C_6_H_9_N_3_O_3_) was purchased from Sigma Aldrich (St. Louis, MO, USA). Relevant physicochemical properties of MNZ are presented in Table 1. Hydrochloric acid (HCl 37%) and sodium hydroxide (NaOH, ≥95%) were obtained from HACH Company (Loveland, CO, USA). All solutions were prepared with ultrapure water (Milli-Q). Poly (vinylidene fluoride-hexafluoropropylene), PVDF-HFP, with a molecular weight of 600,000 g/mol and an HFP content of 12% was supplied by Solvay (Brussels, Belgium). Sigma-Aldrich provided N, N-dimethylformamide (DMF, 99.8%), and TiO_2_ nanoparticles (P25, ≥99.5%) with a surface area ranging from 35 to 65 m^2^/g were supplied by Evonik Industries AG (Essen, Germany).

### 2.2. Synthesis of Ag@TiO_2_ Nanocomposites

The TiO_2_ nanoparticles were functionalised with Ag nanoparticles by the deposition-precipitation method, following procedure ^34^. First, 200 mg of TiO_2_-P25 nanoparticles were dispersed in 40 mL of ultrapure water in an ultrasonication bath for 30 min. Afterwards, the solution was magnetically agitated, and 1.6 mL of a 0.05% AgNO_3_ solution was added. The solution was stirred for 10 min to ensure homogeneous dispersion of the silver nanoparticles. Afterwards, a sodium hydroxide solution (NaOH) was added and mixed for ≈10 min to achieve a pH close to 10. The resulting solution was then centrifuged for 15 min, and the nanocomposite pellet dispersed again in ultrapure water in the ultrasonication bath. This process was repeated twice. The last step was the drying of the nanocomposite in an oven at 80 °C overnight.

### 2.3. Production of Ag@TiO_2_/PVDF-HFP Membrane

Nanocomposites with 10% Ag@TiO_2_/PVDF-HFP were prepared by the solvent-casting method, following the procedure presented in [34]. The concentration of Ag@TiO_2_ nanoparticles in the PVDF-HFP-based membranes assures the optimisation of the combined photocatalytic efficiency and the required mechanical properties [34]. In short, Ag@TiO_2_ nanoparticles were dispersed in DMF by ultrasonication for 3 h to achieve complete dispersion. Afterwards, a PVDF-HFP/DMF concentration of 15:85 *v/v* was prepared and stirred until the complete dissolution of the polymer. The mixed solution was then poured into a glass Petri dish at room temperature for solvent evaporation (≈4–6 days).

### 2.4. Ag@TiO_2_ Nanocomposite Characterisation

The nanocomposite structure and Ag nanoparticles’ distribution over the TiO_2_ surface were evaluated by transmission electron microscopy (TEM, Tecnai T20 from FEI (Gothenburg, Sweden). The nanoparticles were dispersed using a sonication bath, and a drop of the suspension was placed on a copper grid for analysis. Aberration-corrected scanning transmission electron microscopy (Cs-corrected STEM, Gothenburg, Sweden) images were obtained using a high-angle annular dark-field detector in an FEI XFEG TITAN electron microscope operated at 300 kV. Elemental analysis was carried out with an EDX (energy-dispersive X-ray spectroscopy, Gothenburg, Sweden) detector, which performs EDX experiments in the scanning mode.

The crystallographic phases of the pure TiO_2_ and the Ag@TiO_2_ nanocomposites were evaluated by X-ray diffraction using a Bruker D8 DISCOVER diffractometer (Billerica, MA, USA) with incident Cu Kα radiation (40 kV and 30 mA).

Furthermore, UV-Vis reflectance spectroscopy’s optical properties of the pristine TiO_2_ and the Ag@TiO_2_ nanocomposites were assessed using a Shimadzu UV-2501-PC (Kyoto, Japan) set up equipped with an integrating sphere. The spectra were acquired in reflectance mode, and the bandgap was estimated via the Kubelka-Munk theory, Equation (1), and the Tauc plot, represented by Equation (2):

(1) F(R)=(1−R∞)2(2R∞)

where *R_∞_ (R_Sample_/R_BaSO4_*) corresponds to the sample’s reflectance and *F (R)* to its absorbance.

(2) [F(R)hυ]1n versus hυ

where *h* is the Planck constant (6.626 × 10^−19^ J), *υ* is the frequency of vibration, and *n* is the sample transition (indirect transition, *n* = 2).

### 2.5. Ag@TiO_2_/PVDF-HFP Membrane Characterisation

Morphological characterisation of the pure and nanocomposite membranes was assessed by scanning electron microscopy (SEM, NanoSEM e FEI Nova 200 (FEG/SEM), Lincoln, NE, USA) with an accelerating voltage of 10 kV. All samples were coated with a 20-nm-thick gold layer by magnetron sputtering with a Polaron SC502 apparatus.

The nanocomposite polymer crystalline phases were evaluated by Fourier transform infrared spectroscopy (FTIR, Tokyo, Japan) measurements, performed in the attenuated total reflectance (ATR) mode at room temperature with a Jasco FT/IR-4100 apparatus. Analyses were conducted in the spectral range between 4000 and 600 cm^−1^ after 32 scans with a resolution of 4 cm^−1^.

The relative fraction of the electroactive and highly polar *β* phase (*F (β)*) of the polymer was calculated according to Equation (3) [47]:

(3) F(β)=Aβ(KβKα)Aα+Aβ

where *A_α_* and *A_β_* are the absorbances at 766 and 840 cm^−1^, corresponding to the *α* and *β* phase of the polymer, respectively, and *K_α_* (6.1 × 10^4^ cm^2^/mol) and *K_β_* (7.7 × 10^4^ cm^2^/mol) are the absorption coefficients at the corresponding wavenumber.

Differential scanning calorimetry (DSC, Columbus, OH, USA) analysis was carried out with Mettler Toledo DSC 822e equipment between 25 and 200 °C at a heating rate of 10 °C/min under a flowing nitrogen atmosphere. All samples were measured in 30 µL aluminium pans with perforated lids to allow the volatiles’ release and removal.

The wettability of the membranes was assessed through static contact angle measurements. The assays were performed at room temperature with a Neurtek OCA15EC DataPhysics device (Pontevedra, Spain) using ultrapure water (5 µL droplets) as the test liquid. Three measurements were performed for each sample, and the average contact angle was estimated using the digital image.

### 2.6. Photocatalytic Degradation of Metronidazole

The photocatalytic degradation of MNZ was carried out in a glass Petri dish. Using solar irradiation, the assays were carried out in northern Algeria (latitude 36.39°; longitude 2.42° at sea level). The UV intensity of solar radiation was measured with a Pyranometer CMP 11 (Kipp & Zonen, Delft, The Netherlands) with a spectral range from 285 to 2800 nm.

**Figure 1:**
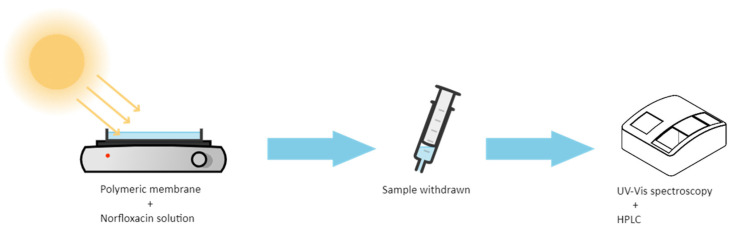
Schematic illustration of the photocatalytic degradation setup and monitoring assays performed.

The Ag@TiO_2_/PVDF-HFP nanocomposite membranes (16 cm × 16 cm × 150 µm) were placed at the bottom of a glass Petri dish with a 250 mL capacity volume. For the photocatalytic assays, 150 mL of MNZ standard solution was poured in the dark into the glass Petri dish containing the membranes at the bottom and stirred for 30 min. After that, the glass Petri dish was placed under magnetic agitation and sunlight irradiation for 5 h, and 3 mL aliquots were withdrawn hourly. Besides the degradation under sunlight irradiation, the photocatalytic degradation of MNZ was also studied by artificial UV irradiation (PHILPS PL-L 24W/10/4P UV lamps, λ_max_ = 365 nm and I = 18.6 W/m^2^). The schematic illustration of the photocatalytic assays setup is presented in Figure 1.

The concentration of MNZ was measured by UV-Vis spectrophotometry (Shimadzu UV1800, λ_max_ = 320 nm) using a 1 cm quartz cell. The photocatalytic efficiency (%) was estimated using the following relation:

(4) $$R\;\left(\%\right)=\frac{\left(C_{0}-C\right)}{C_{0}}\times 100\;$$

where *C*_0_ and *C* are the initial and equilibrium MNZ concentrations (mg/L), respectively.

High-performance liquid chromatography (HPLC) was performed to study the concentration and the degradation mechanism of MNZ using the following parameters: Walters, Waukesha, WI, USA, with a UV detector at 348 nm; a Diamonsil R C_18_ column (5 µm × 250 mm × 4.6 mm ID); mobile phase composed of a combination of acetonitrile and distilled water (30/70, *v*/*v*); data recorded by ChemStation software (B.04, Dayton, OH, USA); flow rate: 1.0 mL/min; and injection volume: 20 μL.

## 3. Artificial Neuro-Fuzzy Inference System Model

An adaptive neuro-fuzzy inference system (ANFIS) is a helpful type of artificial neural network that offers an alternative to the polynomial regression method as a modelling tool [48,49]. Proposed for the first time by Jang et al. in the early 1990s and based on Takagi and Sugeno’s fuzzy interference system, an ANFIS incorporates an artificial neural network (ANN) and fuzzy logic conceptions, and receive the benefits of both in a single frame [50,51]. To describe the general principles, the ANFIS model is defined by an architecture with two input variables (*x*, *y*) and one output (*f*), and the system corresponds to a set of fuzzy IF–THEN rules, expressed as:

Rule 1: If *x* is *A*_1_ and *y* is *B*_1_, Then

(5) $$f_{1}=p_{1}x+q_{1}y+r_{1\;}$$

Rule 1: If *x* is *A*_2_ and *y* is *B*_2_, Then

(6) $$f_{2}=p_{2}x+q_{2}y+r_{2\;}$$

where *A*_1_, *A*_2_, *B*_1,_ and *B*_2_ are the membership functions (MFs) for x and y vectors, respectively; *p*_1_, *q*_1_, *r*_1_, *p*_2_, *q*_2_, and *r*_2_ are the parameters of the output function, and *f* is the weighted mean of the single-rule outputs.

**Figure 2:**
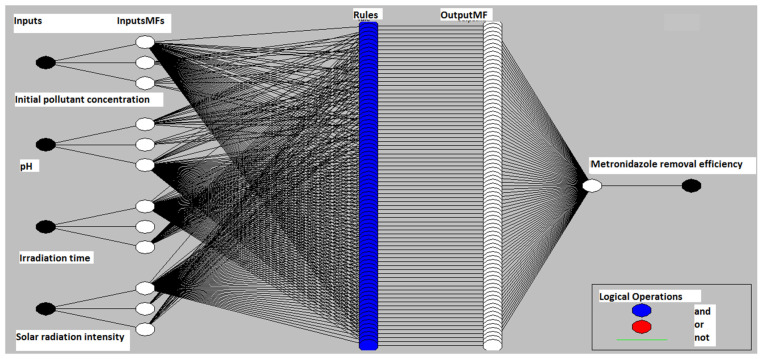
Schematic representation of the five-layer architecture of an artificial neuro-fuzzy inference system (ANFIS) model and the photocatalytic process variables used as inputs.

Five layers compose the architecture of the ANFIS framework, which are outlined in Figure 2. The first layer, the so-called inputs layer, uses the input values and performs the fuzzy formation for each input value. The second layer, or the input MFs, is responsible for performing fuzzy rules.

The role of the third layer (rules layer) is to normalise the membership functions. The fourth layer (output MFs layer) uses the normalised values and concludes the fuzzy rules. The values generated by this layer are defuzzificated and passed on to the last layer to generate the final output [52]. Moreover, the first and fourth layers have adaptive nodes (the training algorithm updates the parameters of these nodes), and the remaining layers have non-adaptive or fixed nodes [53]. Figure 1 shows the ANFIS model structure.

In this study, the ANFIS model was developed in Matlab to predict the degradation efficiency of MNZ by Ag@TiO_2_/PVDF-HFP nanocomposites. The program allows the optimisation of the structure of the ANFIS model in terms of input membership function shape. Three different types of functions were evaluated (trapezoidal, triangular, and Gaussian). The first purpose was to find the most suitable membership function that causes the ANFIS output to match the training data. The ANFIS model’s inputs under consideration were the initial concentration of MNZ, the pH, the irradiation time, and the solar irradiation intensity (Table 2). The removal efficiency of the pollutant was selected as the output.

Therefore, this work’s ANFIS model structure has four neurons in the input layer and one neuron in the output layer. Table 2 summarises the model inputs and their variation range. Identical membership functions for each input neuron were used. For the output neuron, a linear membership function was selected.

## 4. Results and Discussion

### 4.1. Ag@TiO_2_ Nanocomposite Characterisation

**Figure 3:**
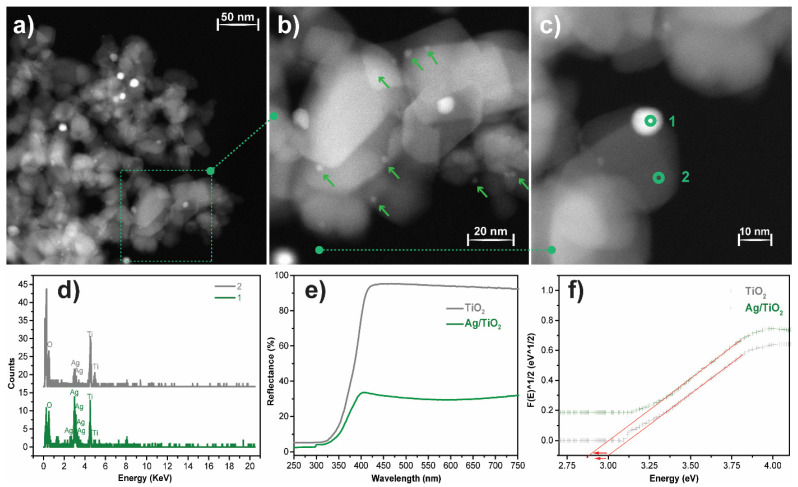
(**a**–**c**) STEM-HAADF-EDX images of Ag@TiO_2_ nanocomposites with different amplifications, indicating the different measured regions: (1) Ag and (2) TiO_2_; (**d**) EDX spectra with elemental identification of regions (1) and (2); (**e**) UV-Vis reflectance spectra of pure TiO_2_ and Ag@TiO_2_; (**f**) estimation of the bandgap for TiO_2_ and Ag@TiO_2_ samples at [F (R)]1/2 = 0.

High-angle annular dark-field scanning transmission electron microscopy (STEM-HAADF, Enschede, The Netherlands) analysis was performed to evaluate the morphology and distribution of Ag nanoparticles over the surface of the TiO_2_ nanoparticles. Figure 3a–c shows round-shaped Ag nanoparticles well-distributed over the surface of the TiO_2_ nanoparticles. Further, STEM micrographs allow for the estimation of the average Ag nanoparticle size and the diameter of the TiO_2_ nanoparticles, being 10 and ≈31 nm, respectively. The elemental analysis of the Ag@TiO_2_ nanocomposite by STEM-HAADF-EDX measurements (Figure 3c,d) is shown in two representative regions: in region one it is shown the Ag predominance over other elements, corresponding to Ag nanoparticles; region two, on the other hand, shows the presence of titanium (Ti) and oxygen (O), identifying the TiO_2_ nanoparticles.

The crystal structure of the TiO_2_ nanoparticles and Ag@TiO_2_ nanocomposites was assessed by X-ray diffraction, and the results are presented in the supplementary material (Figure S1). The characteristic peaks of rutile (27.49°) and anatase (25.3°, 37.8°, and 48.0°) are present in both diffractograms and agree with the literature [26,54]. There are no significant differences between the positions of the characteristic peaks and their intensities. Moreover, Ag diffraction peaks were not observed, related to its low concentration in the nanocomposites and its small size [26,34].

A UV-Vis diffuse reflectance spectrum (DRS) was used to evaluate the optical properties of pristine TiO_2_ and Ag@TiO_2_ nanocomposites, and the results are presented in Figure 3d). According to the figure, TiO_2_ reflects more than 90% of visible-range radiation (400 to 800 nm), and absorbs almost all the radiation in the UV range (<400 nm). This profile is consistent with the photocatalytic activity of TiO_2_ under UV radiation [29]. In the UV range, Ag@TiO_2_ nanocomposites present a similar behaviour to pristine TiO_2_ in the UV spectral range, showing absorption of almost all the radiation. However, in the visible spectrum (400–700 nm), the nanocomposites reflect ≈37% of radiation, >50% less reflection when compared to pristine TiO_2_.

Thus, Ag@TiO_2_ nanocomposites show a reduced reflectance in the visible region, which is related to Ag nanoparticles’ surface plasmon resonance, which is responsible for the higher visible-radiation absorbance [55,56]. The bandgap values were calculated through the Kubelka-Munk equations, as represented in the inset in Figure 3d. The Ag@TiO_2_ nanocomposite shows a lower energy bandgap than pristine TiO_2_ (2.8 eV against 3.0 eV), which is in good agreement with previous reports on silver-based nanocomposites [55,57]. The bandgap energy reduction is related to the absorption of longer wavelengths, and it is a consequence of the formation of intermediate energy levels between the conduction and valence bands [58,59]. Thus, this characterisation indicated the successful preparation of Ag@TiO_2_ nanoparticles and demonstrated their suitability for photocatalytic processes under sunlight radiation.

### 4.2. Ag@TiO_2_/PVDF-HFP Nanocomposite Membrane Characterisation

**Figure 4:**
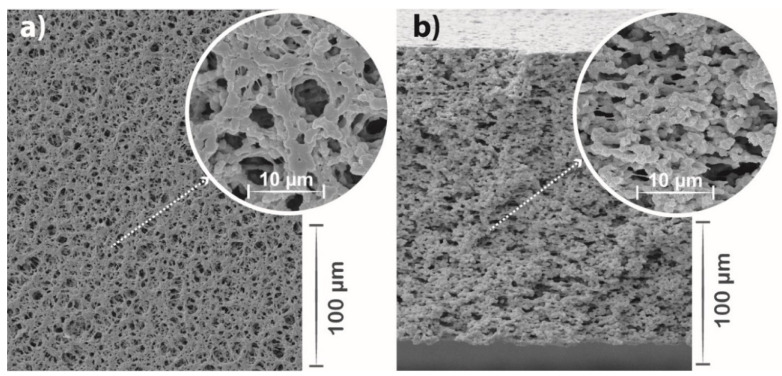
Representative surface (**a**) and cross-section (**b**) SEM micrographs of the 10% Ag@TiO_2_/PVDF-HFP nanocomposite membranes, with the corresponding amplified insets.

SEM images were used to analyse the morphology of the polymeric membranes and nanoparticles dispersion, as represented in the representative images shown in Figure 4.

Figure 4 shows membranes with well-distributed pores along with the matrix, promoted by the liquid-liquid phase separation process and slow solvent evaporation [60]. Micrometric-sized pores are observed, ranging from 1 to 4 µm. It is also noticed that the incorporation of Ag@TiO_2_ nanoparticles into the polymeric matrix does not significantly affect the polymeric matrix properties, also resulting in an interconnected porous microstructure [34], as reported in previous works with PVDF co-polymer nanocomposite membranes [29,53], where the homogeneous distribution of nanoparticles was equally confirmed [48,61].

**Figure 5:**
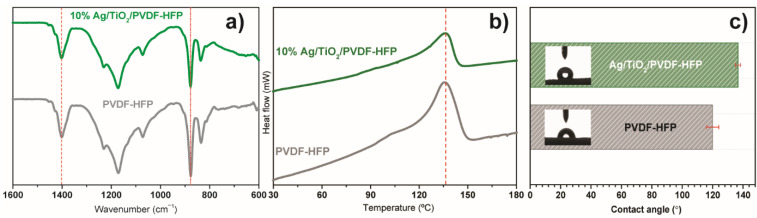
(**a**) FTIR, (**b**) DSC, and (**c**) contact angle measurements of pristine and Ag@TiO_2_/PVDF-HFP membranes.

FTIR spectroscopy was used to determine and quantify the polymeric phases in Ag@TiO_2_/PVDF-HFP membranes (Figure 5a). PVDF-HFP presents the β phase characteristic vibration modes at 840 and 1402 cm^−1^ [62]. Solvent-cast PVDF-HFP membranes are dominated by the electroactive and polar β phase, which is determined by solvent evaporation at low temperatures [63]. The β phase content was estimated by using Equation (1), and the results show that the incorporation of the Ag@TiO_2_ nanocomposite into the PVDF-HFP substrate leads to a decrease in the β phase content from 78 to 57%. This decrease is related to the positive surface charge of TiO_2_ nanoparticles [26], which affects polymer chain conformation during crystallisation through the electrostatic interaction of Ag@TiO_2_ surface charge with the polar C-F bonds of the polymeric chain [64].

DSC allowed the evaluation of the membranes’ thermal transitions, as represented in Figure 5b. Similar thermal behaviour is observed in both samples, with a single endothermic peak at ≈140 °C, indicating the melting of the crystalline phase. The narrow melting peaks are attributed to the homogeneous distribution of the membranes’ PVDF-HFP crystalline phase. Incorporating Ag@TiO_2_ nanoparticles into the polymeric matrix causes a shift in the endothermic peak to 114 °C, triggered by polymer-nanoparticle interactions, leading to destabilisation of the crystalline phase, particularly at the polymer-nanoparticle interface [65].

Additionally, as water remediation applications require an interaction between water and the membrane, it is essential to assess its wettability. The water contact angle measurements (Figure 5c) show that PVDF-HFP and Ag@TiO_2_/PVDF-HFP possess a higher water contact angle than 120° and, therefore, both behave as hydrophobic materials, in agreement with previous works [17,48]. Despite its hydrophobicity, this membrane presents a highly porous structure, confirmed by SEM pictures (Figure 4) and previous works [54], that aids in mitigating its hydrophobic effect by favouring water perculation throughout the pores. Additionally, the incorporation of TiO_2_-based nanoparticles allows for the hydrophobicity of the membranes to be reduced upon irradiation, as the nanoparticles become superhydrophilic [61]. In this context, the herein-performed characterisation allows for the validation of the produced nanocatalyst and nanocomposite membrane, as it is in concordance with previous works [26,34,61], as the Ag@TiO_2_ nanoparticles exhibit visible radiation absorbance. Additionally, these nanoparticles are immobilised into a suitable porous microstructure required for water remediation applications.

### 4.3. Photocatalytic Degradation of Metronidazole

**Figure 6:**
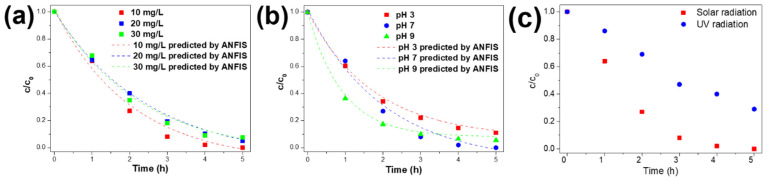
Effect of (**a**) initial MNZ concentration, (**b**) pH, and (**c**) solar as well as UV lamp radiation on the photocatalytic degradation of MNZ.

The study of the Ag@TiO_2_/PVDF-HFP membrane’s photocatalytic properties involved the monitorisation of MNZ absorbance peak during sunlight exposure experiments. The initial concentration (10, 20, and 30 mg/L), the pH (3, 7, and 9), and the type of radiation (solar and UV radiation) were changed in order to study their influence on the photocatalytic process. The results are presented in Figure 6a–c.

Figure 6a shows that the degradation efficiency is not strongly affected by the initial concentration of MNZ, with a degradation efficiency maximum decrease of 7.4% with an increasing MNZ concentration from 10 to 30 mg/L. The MNZ degradation rate is related to the available Ag@TiO_2_ active surface for the production of hydroxyl radicals through the generation of electron-hole pairs [16]. In this study, the concentration of Ag@TiO_2_ nanoparticles was kept constant in an optimised concentration [34]. Consequently, the amount of available hydroxyl radicals remains the same, independently of the MNZ concentration. Increasing the MNZ concentration leads to a decrease in the ratio of HO^•^ radicals/MNZ molecules, which does not affect the degradation efficiency, which remains constant. Thus, the amount of HO radicals generated can degrade MNZ in the solution efficiently.

The pH is one of the most relevant factors influencing the removal of pollutants from water, as it affects the surface charge of pollutants, the predominant property for the effective adsorption of MNZ on Ag@TiO_2_ surfaces [66]. MNZ is protonated at acidic pH values and presents its neutral form in neutral and alkaline conditions [67]. Figure 6b shows that the degradation of MNZ is affected by the pH of the solution, as confirmed by the differences between photocatalytic efficiencies (88.9%, 100%, and 94.5% for pH 3, 7, and 9, respectively. The first hour of the experiment reveals a higher effect of alkaline pH on MNZ degradation (63.6% under pH 9) than degradation at neutral and acidic pH conditions (36.0%, and 39.6% for 7 and 3, respectively). This difference is a consequence of the protonation of MNZ, in acidic pH values, and the possible repulsions between the MNZ-H^+^ species and the Ag@TiO_2_ surface, as the nanocomposites present points of zero charge (PZC) of 6.3, and for pH below PZC it is positively charged [68]. On the other hand, as the surafec of Ag@TiO_2_ is negatively charged at alkaline pH values, the degradation is faster than at acidic pH values. As the increase in pH results in the predominance of neutral MNZ species, the repulsions are weaker or inexistent, and the degradation occurs at a higher rate.

According to Figure 6c, after 5 h under sunlight exposure, the 10% Ag@TiO_2_/PVDF nanocomposite completely degraded MNZ. However, under UV lamp irradiation, the same nanocomposite presents a degradation of ≈ 71%, resulting in a degradation efficiency decrease of 29%. The differences in UV intensity between the UV lamp and UV radiation from the sunlight during the photocatalytic assays, 281 W/m^2^ from UV lamps and 894 W/m^2^ under sunlight radiation, explain the differences in degradation efficiencies. The results show that larger intensities lead to improved degradation efficiencies due to the activation of a higher number of electron/hole pairs [39]. This relationship is consistent with previous studies using TiO_2_ for photocatalytic applications under sunlight irradiation [16,69].

Despite the reduced number of works focused on photocatalytic treatments against MNZ, some can be found, mainly in slurry systems (Table 3).

All the presented materials present relatively similar efficient degradations in the 80–99% range, and despite the description of experimental conditions, many other experimental parameters do not allow direct comparisons between works. Most of the presented works are performed with expensive energy (UV) and slurry systems (nanoparticles in suspension), making the process difficult to be scaled for applications. Despite the potential limitations of photocatalyst-immobilised systems (reduced surface area, mass transfer limitations, and reduced radiation harvesting), our work proves that, even if it requires more exposure time when compared to MnWO_4_/Bi_2_S_3_ [74], complete degradation of MNZ is achieved. Moreover, Ag@TiO_2_/PVDF-HFP membranes show the most significant advantages: their utilisation of sunlight, their reusability (see the following section), and their avoidance of contamination through nanoparticle lixiviation into water bodies.

### 4.4. Reusability of the Nanocomposite

**Figure 7:**
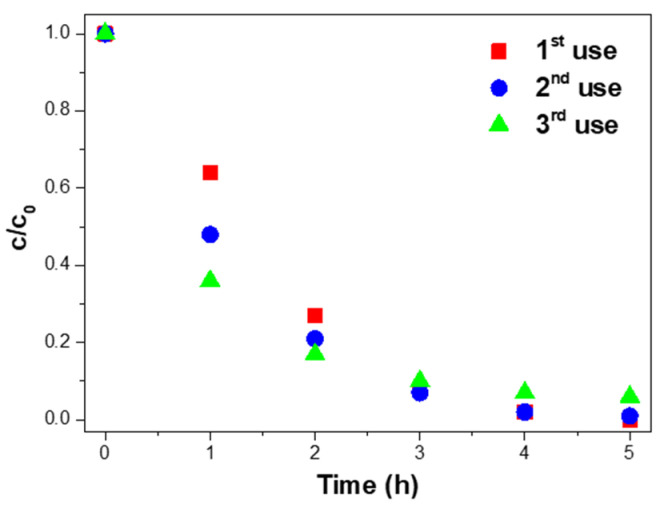
Photocatalytic degradation of MNZ (10 mg/L) with 10% Ag@TiO_2_/PVDF–HFP membranes in three consecutive uses, under 5 h of sunlight exposure in each experiment.

Three consecutive uses assessed the reusability of the 10% Ag@TiO_2_/PVDF-HFP nanocomposites. After each use, the nanocomposite was washed with UP and dried at room temperature. Later, for a new use, the membrane was placed at the bottom of the glass Petri dish with a new MNZ solution under the same experimental conditions. The results are presented in Figure 7.

Figure 7 shows the complete degradation of MNZ after the first use of the 10% Ag@TiO_2_/PVDF-HFP membranes. In the second and third uses, 99.9% and 94.0% of MNZ was degraded, representing a maximum of ≈6.0% efficiency loss after three uses. Although the efficiency loss is insignificant, it may be explained by the detachment of Ag@TiO_2_ nanoparticles from the PVDF-HFP membranes during use or washing processes [75,76]. Further, the efficiency loss can also be related to small amounts of MNZ accumulated on the nanocomposite membrane surface after each use, and as a consequence, a decrease in active sites in subsequent uses [77].

### 4.5. Mineralisation and Degradation Mechanism

**Figure 8:**
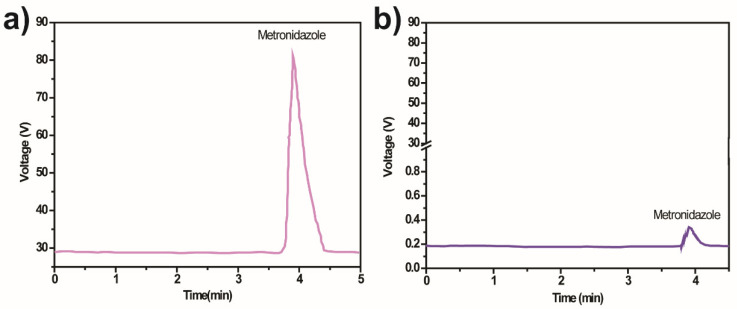
HPLC chromatogram of MNZ (C_i_ =10 mg/L) samples (**a**) before and (**b**) after photocatalytic degradation by 10% Ag@TiO_2_/PVDF-HFP under 5 h of sunlight irradiation.

The mineralisation evaluation is essential to confirm the degradation of MNZ monitored with UV-Vis spectrophotometry and to assess the possible formation of intermediate byproducts during a photocatalytic degradation process. The production of these intermediates and the degradation mechanism was assessed through HPLC (Figure 8).

The chromatogram analysis reveals differences before and after the degradation of MNZ by the 10% Ag@TiO_2_/PVDF-HFP membranes. This difference indicates no formation of intermediates after the 5 h of the degradation process, and that the degradation yields H_2_O, CO_2_, and NH_4_^+^.

**Figure 9:**
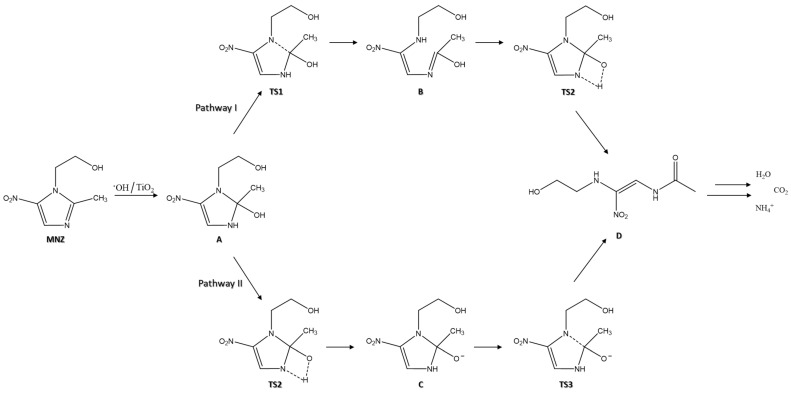
Schematic representation of two possible paths for the opening of the imidazole ring in the degradation mechanism of MNZ (adapted from [78]).

According to previous work [78], the degradation mechanism of MNZ begins with the adsorption of this molecule to the Ag@TiO_2_ surface. A H atom on the branch of the imidazole ring and the metronidazole methyl group can form hydrogen bonds to the O atoms of Ag@TiO_2_ [47]. Additionally, both hydroxyl and nitro O atoms of MNZ can adsorb on the nanocomposite Ti atom [79]. Electrons and holes are excited when photons irradiate the Ag@TiO_2_ surface with energy higher than the bandgap energy, and OH radicals are formed to degrade the MNZ molecule [80]. The opening of the imidazole ring is the fundamental step of this antibiotic degradation. Two possible mechanisms for this step are presented in Figure 9.

The first step is the oxidation of MNZ in the presence of Ag@TiO_2_ molecules, to originate subproduct A. The next step is to break the C (2)–N (1) bond and transfer protons into the imidazole ring [81]. According to pathway I, the C (2)–N (1) bond is broken, and the H (1) atom of the hydroxyl group is transferred to form a bond with the N (3) atom of the imidazole ring (TS1). Consequently, an enol structure is formed on the C (2) atom of MNZ (subproduct B). Subsequently, the hydroxyl group H (1) atom directly undergoes a transition state from a four-membered ring structure when transferred to the N (3) atom (TS2). Then, the final product of the ring-opening step is formed (subproduct D). According to pathway II, firstly, the transfer of the H (1) atom from the hydroxyl group to the N (3) atom of the imidazole ring through a transition state of the four-membered ring (TS2). After the H (1) atom transfer to the N (3) atom, the subproduct C is formed. In this stage, the O (1) atom is adsorbed onto the Ag@TiO_2_ surface through electrostatic interactions [78]. As the bond lengths of N (3)–C (2)–O (1) shorten, the C (2)–N (1) bond starts to break (TS3). Then, the C (2)–N (1) bond is broken to form the subproduct D [78]. The last step is the complete mineralisation of the final product into H_2_O, CO_2_, and NH_4_^+^.

Different approaches can lead to several activation steps for MNZ degradation, so alternative pathways may be considered. One alternative pathway is the degradation by solar photo-Fenton processes, as proposed by Ammar, H. B. et al. [82].

### 4.6. ANFIS Results and Discussion

**Figure 10:**
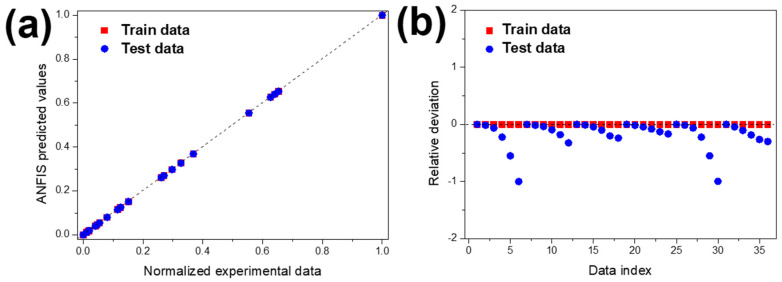
(**a**) Regression plot of the ANFIS model and (**b**) relative deviation for test and train data.

The predictability of MNZ degradation was investigated by using the developed ANFIS model. In determining the ANFIS model’s inputs, the MNZ degradation efficiency was directly related to experimental parameters such as initial contaminant concentration and pH. For both experimental conditions, three data points were used, and experimental results were used to train the ANFIS model. Figure 10 shows the ANFIS-predicted results against normalised experimental data for train and test values.

As represented in Figure 6a,b, concerning MNZ concentration and pH, the experimental and the ANFIS-predicted MNZ degradation curves are similar, which means that the training data (experimental results) and the predicted data obtained by the ANFIS model are in good agreement. Figure 10a shows the predicted values against normalised experimental data and test in addition to train data, respectively. The accumulated values close to the *y* = *x* line indicates the suitable implementation of the ANFIS model. This indicates that the ANFIS model’s accuracy is acceptable, and that there is a good agreement between experimental and predicted results for MNZ degradation. The relative deviation plot shown in Figure 9 was used to determine the model structure’s capability. For a suitable model structure, all the relative deviation values must be placed between −1 and 1 [83]. The accumulated relative deviation values close to the y = 0 line indicates the developed model structure’s accuracy [84]. Statistical parameters such as mean squared error (MSE), root mean square error (RMSE), mean absolute error (MAE), mean absolute percentage error (MAPE), and correlation coefficient have been used to evaluate the accuracy of the ANFIS model. These values were calculated according to previous works [83,84]. The parameters are presented in Table 4.

The MSE, RMSE, and MAE statistical parameters evaluate the ANFIS model’s accuracy, and lower values suggest a good fit for the model [85]. Low values were obtained for these parameters, which indicates the suitability of the developed model. Otherwise, the R^2^ value should be proximate to unity for an excellent correlation between test and predicted results [86]. The obtained R^2^, close to 1, expresses the high capability of the developed model. Taking all of this into account, the ANFIS model presents high accuracy in predicting MNZ degradation efficiency. Considering initial MNZ concentration and pH dependence on this contaminant degradation efficiency, this model could forecast different performances.

## 5. Conclusions

Developing highly efficient catalysts for water and wastewater treatment under sunlight irradiation is a promising approach to address the urgent demand for water remediation, particularly for remediation of emerging pollutants such as MNZ. Thus, composite membranes based on Ag@TiO_2_ and PVDF-HFP have been prepared and characterised, and their photocatalytic activity has been evaluated for the degradation of MNZ under sunlight radiation.

Ag-nanoparticle-decorated TiO_2_ nanocomposites allowed a bandgap energy of 2.8 eV to be obtained, which is lower than the bandgap energy of pristine TiO_2_ (3.0 eV). The prepared 10% Ag@TiO_2_/PVDF-HFP nanocomposite membranes presented a porous microstructure with well-distributed pores. The synthesised composite membrane’s photocatalytic activity was evaluated by varying relevant operational parameters, such as the initial concentration of the pollutant, the pH, and the intensity of irradiation, and theoretically evaluated by an artificial neuro-fuzzy inference system (ANFIS), allowing for the optimisation of parameters for MNZ photocatalytic degradation under solar radiation. The maximum degradation efficiency of 100% was achieved with an initial MNZ concentration of 10 mg/L, at a pH of 7, and after 5 h of solar irradiation. After three consecutive uses, the degradation efficiency proved consistent, with an efficiency loss of 6%. The ANFIS model proved to be a suitable method to predict MNZ degradation efficiency, as the comparison between experimental and predicted results were similar and the calculated statistical parameters confirmed its accuracy for this application.

Thus, the prepared 10% Ag@TiO_2_/PVDF-HFP membranes are suitable for water remediation of MNZ and related emerging pollutant contamination under solar irradiation.

## Figures and Tables

**Table 1:** Relevant characteristics of metronidazole (MNZ) [45,46].

Molecular Weight (g/mol)	Molecular Structure	Solubility in Water (mol/L)	pK_a1_	pK_a2_
171.15	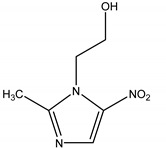	0.041	2.58	14.44

**Table 2:** Process variables and their variation range.

Process Variables	Range
Initial concentration of metronidazole (mg/L)	10–30
pH	3–9
Irradiation time (h)	0–5

**Table 3:** Comparison of MNZ degradation efficiencies by different photocatalysts.

Photocatalyst	Degradation	Experimental cCnditions	Reference
TiO_2_/Fe^3+^	97%	UV radiation = 125 W; C_i_ = 80 mg/L; time: 2 h	[70]
TiO_2_/Fe_2_O_3_/GO	97%	UV radiation = 15 W; C_i_ = 10 mg/L; time: 2 h	[71]
CuO	98.4%	UV radiation = 55 W; C_i_ = 50 mg/L; time: 1 h	[72]
MnFe-LDO-biochar	98%	UV radiation = 20 W; C_i_ = 20 mg/L; time: 2 h	[73]
MnWO_4_/Bi_2_S_3_	79.8%	Visible radiation = 400 W/m^2^; C_i_ = 20 mg/L; time: 3 h	[74]
Ag@TiO_2_/PVDF-HFP	100%	Solar radiation = 800 W/m^2^; C_i_ = 10 mg/L; time: 5 h	This work

**Table 4:** Statistical parameters of the developed ANFIS model.

Statistical Parameters	Value
MSE	0.002
RMSE	0.044
MAE	0.174
MAPE (%)	2.424
R^2^	0.98

## Data Availability

Not applicable.

## Supplementary Materials

The following are available online at https://www.mdpi.com/article/10.3390/polym13213718/s1, Figure S1: X-ray diffraction patterns of pristine TiO_2_ and Ag-TiO_2_ nanocomposite and identification of the representative peaks for anatase (A) and rutile (R) phases.
