# Supplementary material for: Reusable Ag@TiO2-Based Photocatalytic Nanocomposite Membranes for Solar Degradation of Contaminants of Emerging Concern
Source: Polymers (Basel). 2021 Oct 28;13(21):3718. doi: 10.3390/polym13213718 (PMC8587559; doi:10.3390/polym13213718)
Supplement: Supplementary file 1 [file polymers-13-03718-s001.zip › polymers-1395411-supplementary.pdf]

Supplementary material

Reusable Ag@TiO2 Based Photocatalytic Nanocomposite Membranes for Solar Degradation of Contaminants of Emerging Concern

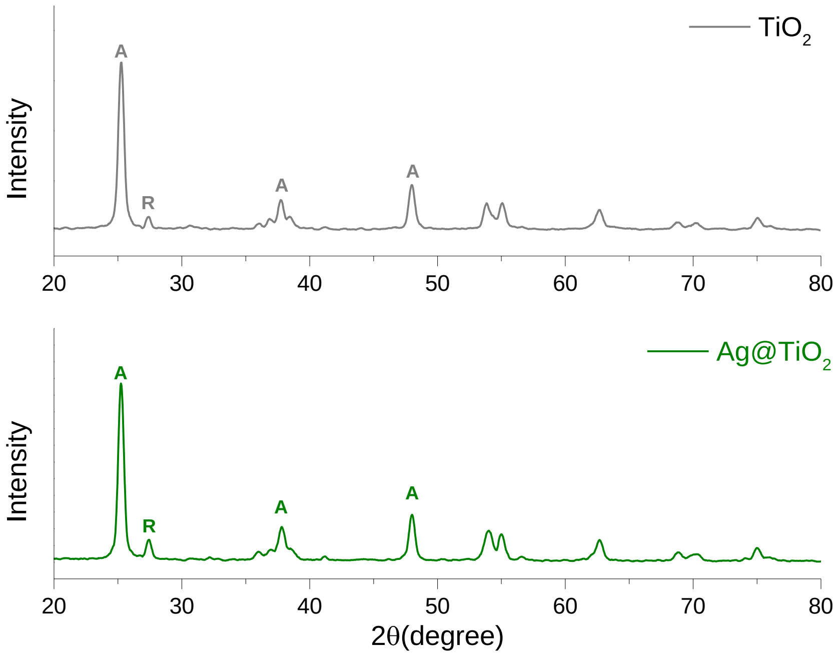

Figure S1. X-ray diffraction patterns of pristine TiO_2 and Ag-TiO_2 nanocomposite and identification of the representative peaks for anatase (A) and rutile (R) phases
